# Cytotoxic, Apoptotic and Genotoxic Effects of Lipid-Based and Polymeric Nano Micelles, an In Vitro Evaluation

**DOI:** 10.3390/toxics6010007

**Published:** 2017-12-30

**Authors:** Fatemeh Bahadori, Abdurrahim Kocyigit, Hayat Onyuksel, Aydan Dag, Gulacti Topcu

**Affiliations:** 1 Department of Pharmaceutical Biotechnology, Faculty of Pharmacy, Bezmialem Vakif University, Fatih, Istanbul 34093, Turkey; fatemehbahadori@hotmail.com (F.B.); gul@topcular.net (G.T.); 2 Department of Medical Biochemistry, Faculty of Medicine, Bezmialem Vakif University, Istanbul 34093, Turkey; 3 Department of Biopharmaceutical Sciences, University of Illinois at Chicago, Chicago, IL 60612, USA; hayat@uic.edu; 4 Department of Pharmaceutical Chemistry, Faculty of Pharmacy, Bezmialem Vakif University, Fatih, Istanbul 34093, Turkey; aydandag@gmail.com

**Keywords:** cytotoxicity, genotoxicity, lipid-based micelles, Nano Drug Delivery System, polymeric micelle, targeted cancer therapy

## Abstract

Self-assembly systems (SAS) mainly consist of micelles, and liposomes are the classes of Nano Drug Delivery Systems with superior properties compared to traditional therapeutics in targeting cancer tumors. All commercially available nano-formulations of chemotherapeutics currently consist of SAS. According to our knowledge, a specific toxicity comparison based on material differences has not yet been performed. The purpose of this study was to evaluate and compare the toxicity of two SAS consisting of Sterically Stabilized Micelles (SSM) made of a lipid-based amphiphilic distearoyl-sn-glycero-phosphatidylethanolamine-polyethylene glycol (PEG)-2000 and a polymeric micelle (PM) consisting of Y-shape amphiphilic block copolymer, synthesized using poly ε-caprolactone and PEG. The mechanism of cytotoxicity and genotoxicity of micelles on L-929 healthy mouse fibroblast cells was assessed using Sulforhodamine-B, WST-1, Acridine Orange/Ethidium Bromide and alkaline single-cell gel electrophoresis assays. Results showed that SSM in conc. of 40 mg/mL shows very low cytotoxicity at the end of 24, 48 and 72 h. The DNA damage caused by SSM was much lower than PM while the latter one showed significant toxicity by causing apoptosis with the ED50 value of 3 mg/mL. While the DNA damage caused by SSM was ignorable, some DNA chain breaks were detected on cells treated with PM.

## 1. Introduction

In recent decades Nano Drug Delivery Systems (NDDS) have become one of the most striking areas of biotechnological study. NDDS are most commonly used to enhance the efficacy and safety of conventional drugs. One of the most important applications of NDDS is targeted cancer therapy. This application is based on the irregular packing of vein endothelial cells at the tumor side. Vein endothelial cells are regularly ranked in healthy tissues and the gap between them is measured as less than 5 nm. However, in case of tumorigenesis this gap increases to 200–750 nm because of local inflammation, which is generally associated with all neoplasmic structures. This property is called Enhanced Permeability and Retention (EPR) [1]. EPR effect allows the NDDS to penetrate only to the tumor site where the payload will be released effectively. In this way, the chemotherapeutic agent shows its effect only at the cancer tissue and the treatment with lower clinical doses becomes possible. More importantly, this strategy provides delivery of the high dose of chemotherapeutic agent to the tumor side which results in better results from the first application, protecting healthy cells from its harmful effects.

The most commonly used NDDS in targeted cancer therapy are “self-assembly systems” (SAS) made of amphiphilic materials. These materials are consisted of hydrophilic and hydrophobic blocks, which are able to form bilayered vesicles or monolayered micelles in aqueous media, depending on their structure [2]. In these systems, the hydrophobic blocks collapse together, exposing the hydrophilic blocks to the outer aqueous media. The hydrophobic compartment incorporates with hydrophobic drug molecules, while the hydrophilic compartment on the surface is responsible for the circulation of the drug delivery system in the blood stream. 

SAS are superior to other NDDS because of high drug loading capacity, easy assembling in aqueous media with sizes less than 100 nm, applicable large-scale manufacturing technologies and the numerous biocompatible and biodegradable amphiphilic materials that are available for their production [3]. Hafner et al. have successfully reviewed and reported the commercially available nano-formulations in Europe [4], the dominant quantity of which consist of SAS, and numerous authors have reported the enhanced efficacy of drugs encapsulated in liposomes and micelles in the frame of scientific research or in the commercially available formulations [5,6,7].

Not only SAS but also all NDDS and all nano engineered materials are considered for their possible toxicities by supervising authorities such as the U.S. Food and Drug Administration, FDA [8]. 

“FDA’s Approach to Regulation of Nanotechnology Products” [9] describes two points of consideration. These points address 1) particle dimensions and 2) dimension-dependent properties or phenomena of materials. These two criteria reveal whether a regulated product is involved with the FDA’s application of nanotechnology or not. To this end, the FDA prepared a scientific plan in 2013 called “2013 Nanotechnology Regulatory Science Research Plan” by which the FDA aims to provide coordinated leadership to address key scientific gaps in knowledge, methods, or tools needed to make regulatory assessments of nano products [10]. 

In this context, the National Center for Toxicological Research (NCTR), the most important center of the FDA involved in the regulation of different products, is developing analytical tools and procedures to quantify nano-materials in complex matrices and conducting toxicity studies on nano-materials. The NCTR reports that its areas of research include evaluating the genotoxicity of engineered nano-particles or materials that are used in various FDA-regulated applications ranging from tissue engineering to cancer targeting. Accordingly, the NCTR reports that it will organize collaborative and interdisciplinary research to address product characterization and safety [11]. 

Meanwhile, the CDER (Center for Drug Evaluation and Research Nanotechnology Programs), an organization operating under FDA, has established research projects to address the following:

- “Identify the limitations of current test methods to assess the quality and safety of nano-particle-based therapeutics; and
- Evaluate the application of nanotechnology on product characteristics, including stability and content uniformity” [12].

Based on all the above-mentioned considerations and requirements, toxicity assessment of all synthesized NDDS is of importance for production of a nano engineered material. Any scientific study in the field of drug development and innovation could not be acceptable without taking the end product requirements into account. As can be seen above, the FDA and its related organizations have already started applying specific limitations to nano-engineered products and are planning to enhance toxicity surveillances in this area based on results obtained by scientific research.

Recently, we have reported novel lipid and polymer-based NDDS and showed their superior properties when loaded with drugs compared to conventional drugs [13,14]. Our studied lipid-based micelles consisted of Poly Ethylene Glycole conjugated (PEGylated) phospholipid 1,2-distearoyl-*sn*-glycero-3-phosphatidylethanolamine-*N*-[methoxy (polyethylene glycol)-2000] (DSPE-PEG2000). These PEGylated lipid micelles, also called “Sterically Stabilized Micelles” (SSM) are well-established NDDS and are used in our laboratory in formulation of several hydrophobic anti-cancer drugs due to their long-circulating, biocompatible and biodegradable properties [15]. The polymeric micelles (PM) were synthesized by preparation of a Y-shape amphiphilic block copolymer consisting of hydrophobic poly (ε-caprolactone) (PCL) and hydrophilic poly (ethylene glycol) (PEG). Both PCL and PEG are very well known materials for their biocompatible and biodegradable characteristics. In both studies Vinorelbine, a chemotherapeutic agent which is clinically used in treatment of non-small cell lung cancer, was uploaded to the hydrophobic core of synthesized micelles. Our previous studies showed that DSPE-PEG 2000 and PCL-PEG-loaded Vinorelbine shows ≅ 7 and 5 times enhanced cytotoxic activity respectively compared to free Vinorelbine at 72 h in vitro [13,14]. 

In this study, we compared these two amphiphilic materials to understand whether these materials are safe in terms of cytotoxicity and genotoxicity, and whether they are compatible to be developed as NDDS in clinical applications or not. As is well known, the phospholipid part of SSM is a naturally occurring material and PCL is a completely synthetic one. Since the PEG part is the common component of these amphiphilic materials, we hypothesize that this comparison will symbolize the toxicity of natural and synthetic materials. Superior efficacy and better toxicity profile would make good bases to establish further steps in clinical assays.

## 2. Materials and Methods

### 2.1. Materials

Poly(ethylene glycol-2000)-conjugated distearoylphosphatidylethanolamine (DSPE-PEG 2000) was obtained from Lipoid Lipids (MW2810, Cat # PE 18:0/18:0-PEG2000, Lot # 882032-01/907), Heat-inactivated fetal calf serum (FCS), Dulbecco’s modified Eagle medium (DMEM), 2′, 7′-dichloro-dihydrofluorescein-diacetate (CM-H2DCF-DA), penicillin-streptomycin and ethidium bromide (EB) were purchased from Sigma-Aldrich (Seelze, Germany). All other reagents used were of analytical grade unless otherwise stated. Phosphate Buffer saline (1× PBS, pH = 7.4) was prepared by dissolving 8 g NaCl, 0.2 g KCl, 1.44 g Na_2_HPO_4_, 0.24 g KH_2_PO_4_ in 800 mL of distilled H_2_O and pH was adjusted to 7.4 with HCl. Final volume was fixed to 1 L by adding purified H_2_O and subsequently sterilized by autoclave.

#### Synthesis and Analysis of Purity of PCL_2_-PEG

**Figure 1:**
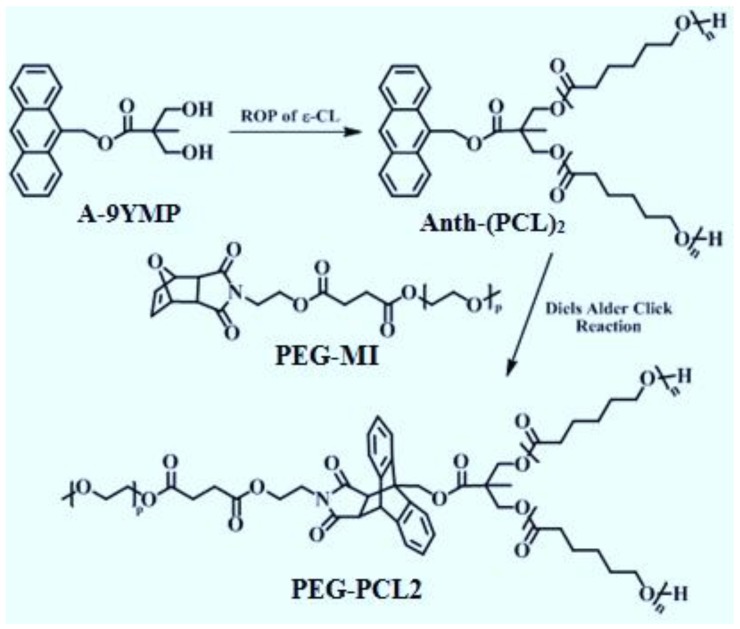
Synthesis of Y-shaped amphiphilic PEG-PCL_2_ in order to prepare polymeric micelles. The figure is adopted from our previously published data [13].

Y-shaped PCL_2_-PEG was synthesized via Diels-Alder click reaction of a furan-protected maleimide end-functionalized PEG (PEG-MI) with an anthracene end-functionalized PCL (Figure 1). A procedure for the synthesis of anthracen-9-ylmethyl 3-hydroxy-2-(hydroxymethyl)-2-methylpropanoate (A-9YMP), was described in our published procedure [16]. Anth-(PCL)_2_ was prepared by ROP of ε-CL (5.0 mL, 0.047 mol) in bulk using tin(II)-2-ethylhexanoate as a catalyst and A-9YMP (0.30 g, 0.94 mmol) as an initiator at 110 °C for 9 h. The details of this reaction and the procedure needed for the precipitation of product is given in our previously published data [13]. In present study all steps were followed as the same and the ^1^H-NMR analysis of obtained Y-shaped amphiphilic material was carried out to insure the purity of the obtained material. The summary of the ^1^H-NMR spectra and the chemical shifts of the important functional groups are given in Table 1.

### 2.2. Instruments

Particle size measurements were made using Malvern ZEN 3600 Zetasizer, Enigma Business Park Grovewood Road U K. Spectrophotometric measurements were made at Varioskan Flash Multimode Reader, Thermo Scientific, Chicago, IL, USA. Fluorescence microscope assays were made at Leica DM 1000, Wetzlar, Germany.

### 2.3. Methods

#### 2.3.1. Preparation of Nano-Formulations

The concentration chosen for preparation of empty nano-micelles in this study was based on previously reported data [14]. It has been shown that the viscosity of SSM solution shows an increase above 15 mM (≅ 42.15 mg/mL) [17]. Meanwhile PMs start precipitating above 50 mg/mL [13]. Based on the above data we prepared samples of 40 mg/mL from both SSM and PMs to make a rational toxicity comparison. It is noteworthy that this comparison is *w*/*v* based. Both micelles are above their Critical Micelle Concentration (CMC). However, we assume that the number of micelles/mL are different from each other in SSM and PM.

Sterically Stabilized Micelles (SSM) were prepared using co-precipitation and reconstitution [18]. 40 mg poly (ethylene glycol-2000)-grafted distearoylphosphatidylethanolamine (DSPE-PEG (2000)), was dissolved in 1 mL methanol. The solvent was removed under vacuum to obtain dry film. The film was then dried more in vacuum desiccator over night to remove any possibly remaining amount of solvent. Micelles were prepared by rehydration of the dried film using 1× PBS buffer, pH 7.4. The micellar solution was then flushed with argon (to prevent oxidation of the lipid chain), sealed, and equilibrated for 2 h at room temperature. 

For polymeric micelles (PM) 40 mg amphiphilic polymer was dissolved in DMF and stirred overnight. Water was added drop wise to this mixture to obtain the final ratio of 67/33 (water/DMF). The mixture was stirred for one more night and dialyzed against double distilled water [14]. The water was exchanged at first, second, third, sixth and twelfth hours [19,20,21,22]. Obtained micelles were filtered, lyophilized and re-hydrated using 1× PBS, pH = 7.4. 

It is noteworthy that the concentrations used in this study to evaluate the healthy cell toxicity are much higher than what is being used to carry the therapeutic amounts of drug molecules. 0.0067 mg (1 mM) SSM/1 mL is enough to carry the therapeutic dose of chemotherapeutic drugs [18,23] and the amount of PM used in vitro is ≈0.028 mg/mL. 

#### 2.3.2. Particle Size Distribution and Zeta Potential Assays

All samples were measured in triplicate. The sizes of lipid and polymer-based nano micelles were measured directly on Zetasizer Nano ZS (model ZEN 3600; Malvern Instrument, Inc., London, UK) at 25 °C. Default setting on the Zetasizer Nano ZS was used, i.e., refractive index, absorption, the dispersant used was water and measurement angle was 173. Measurements were repeated 5 times, 3 min each and Data were analyzed by number, volume and intensity distributions [24]. Zeta potential was calculated by application of Henry Equation [25]. According to our experiences, in case of presence of traces of amorphous aggregates in the system, measurements based on the volume and intensity distributions reveal these aggregates better than the ones based on number distribution. Therefore, all above mentioned measurements conducted to make sure the obtained toxicity results are caused by the micelles, not aggregates. 

The size of nano-micellar drug delivery systems in culture media was measured in order to ensure any change in size of particles in vivo*.* For this supposed, 1 mL of SSM or PM (at the highest concentration chosen for cytotoxicity assay) in PBS was added to 9mL of complete culture media (DMEM, 10% FCS, 100 U/mL of penicillin and 100 ng/mL of streptomycin). This ratio is equal to the ratio of components at each well of cell culture. After slow pipetting, the mixture was incubated at 5% CO_2_ atmosphere at 37 °C for 2 h. At the end of this period the size of nano-particles were measures using zetasizer Nano ZS.

#### 2.3.3. Cell Culture and Maintenance

NCTC clone 929 [L929, derivative of Strain L] mouse healthy fibroblast cells were obtained from American Type Cell Culture Collection (ATCC^®^ CCL1™). Behavior of L-929 cell line upon contact with different materials is accepted as indicator of that of healthy tissues and currently numerous papers report cytotoxicity assays using these cell line [26,27,28,29,30]. Cells were cultured in DMEM equilibrated with 5% CO_2_ atmosphere at 37 °C. The medium was supplemented with 10% FCS, 100 U/mL of penicillin and 100 ng/mL of streptomycin. The number of viable cells was estimated by trypan blue exclusion test. Cells were plated in 96 well plates in number of 5 × 10^3^ cells/well. The day after medium was removed, 90 µL of complete medium and 10 µL of SSM and PM formulations in PBS was added to wells. The highest concentration of formulations was 40 mg/mL (as mentioned above) (final con. 40 mg/mL) and 6 consecutive dilutions consisted of 20, 10, 5, 2.5, 1.25 and 0.625 mg/mL was prepared using PBS as solvent. Control groups with 10 µL of PBS and 100 µL of complete media also were added. Each sample was evaluated in triplicate. Samples incubated for 24, 48 and 72 h at separate plates [31]. 

#### 2.3.4. Cytotoxicity Assay

Cytotoxic activities of SSM and PM were studied using two different assays. Sulforhodamine B (SRB) and WST1. We chose these two methods because their mechanism of action indicates two different behavior of the living cells. SRB is a measurement method of the protein amount of living cells, while WST-1 shows the functionality of mitochondrial respiratory chain. 

##### SRB

At the end of each incubation period, living cells were fixed to wells by adding 100 µL of 20% trichloroacetic acid and incubating for 1 h at 4 °C. The wells then were washed with deionized water, dried under air flow and each well stained with 100 µL of SRB (0.4% SRB in 1% acetic acid) for 30 min in room temperature. 1% acetic acid was used to wash the plates for 3 times and 200 µL/well of 10 mM Tris buffer was added to re-solubilize the stained cells. The viability ratio of cells treated with NDDS compared to non-treated control cells were calculated by measuring their optical density at 515 nm using microplate reader (Varioskan Flash Multimode Reader, Thermo Scientific, Chicago, IL, USA). PBS which was the only vehicle used in this assay, was used as control. These values were then expressed as “%control cell growth” [32] calculated using nonlinear regression analysis (percent survival vs. concentration). 

##### WST-1

At the end of each incubation period, the medium were collected from wells, 90 µL of fresh medium was added to each well and 10 µL of WST-1 reagent was added to each well to obtain a final dilution of 1:10. Well-plates were incubated at 37 °C for four h. Optical density of sample bearing wells were measured at 450 nm with a reference wavelength of 640 nm a multi plate reader (Varioskan Flash Multimode Reader, Thermo Scientific, Chicago, IL, USA), which, is directly correlated with the living cell number, accepting the cell number of non-treated wells as 100. Since the micelles were prepared in PBS, it was used as negative control. The ratio of sample wells to non-treated control cells were reported as cell viability [33,34] and consequently ED_50_ values were calculated. 

#### 2.3.5. Genotoxic Activity Assays (Comet Assay)

##### Sample Preparation

It is accepted that DNA damage is important when the repair mechanism of the cell does not function properly and DNA damage ends with cell death. Therefore, we performed the DNA damage assay based on the above mentioned cytotoxicity assays using the concentration range and ED_50_ values for NDDS after 72 h. The ED_50_ value for PM was calculated as 0.29 mg/mL in both SRB and WST-1 assays. One concentration below ED_50_ and two concentrations above it consisted of 1, 0.5 and 0.25 mg/mL, were chosen to evaluate the DNA damage. The ED_50_ values for SSM was not obtained by SRB and WST-1 assays, even at the highest concentration of nano particles studied. In this case, three highest concentrations 4, 2 and 1 mg/mL were chosen to be tested. 

##### Cell Culture

The alkaline single-cell gel electrophoresis assay (Comet Assay) was used to assess the potential DNA damage caused by NDDS on L-929 cells. The method used was slightly modified method previously reported by Singh et al. [35,36]. Cells were seeded at 6 well plates (1.2 × 10^5^ cells/well). The day after the media was removed and replaced with 900 µL of complete media and 100 µL of SSM or PM samples to obtain 1/10 dilution ratio. At the end of 24, 48 and 72 h. of incubation periods, cells were harvested using a cell scraper, centrifuged and re-suspended in 1 mL of PBS. 

##### Comet Assay

Ten µL of re-suspended cells were placed into Eppendorf tubes and mixed with 75 μL of 0.6% low melting agarose (LMA) and added to the slides pre-coated with 1% normal melting (NM) agarose. Slides were left for 5 min at 4 °C to allow the agarose to solidify. Then the slides immersed in cold (4 °C) lysing solution (2.5 M NaCl, 100 mM Na EDTA, 10 mM Tris–HCl, pH 10–10.5, 1% Triton × 100 and 10% DMSO) for at least 1 h. Slides were removed from lysis solution, washed with cold PBS and placed in an electrophoresis tank horizontally side by side. DNA was unwind using 300 mM NaOH and 10 mM Na_2_EDTA (pH 13.0) for 30 min. After unwinding, electrophoresis was run at 300 mA for 25 min at 4 °C under minimal illumination to prevent further DNA damage. The slides were washed three times with a neutralization buffer (0.4 M Tris, pH 7.5) for 5 min at 4 °C and then treated with ethanol for another 5 min before staining. Dried microscope slides were stained with ethidium bromide (2 μg/mL in distilled H_2_O; 70 μL/slide) covered with a coverslip and analyzed using a fluorescence microscope (Leica DM 1000, Wetzlar, Germany) at a 200× magnification with epiflourescence equipped with a rhodamine filter (with an excitation wavelength of 546 nm; and a barrier of 580 nm). A hundred cells were randomly scored by eye in each sample on a scale of 0–4 based on fluorescence beyond the nucleus. The scale used was as follows: 0, no tail; 1, comet tail <half the width of the nucleus; 2, comet tail equal to the width of the nucleus; 3, comet tail longer than the nucleus; 4, and comet >twice the width of the nucleus. The individual scoring of the slides was blind, using coded slides. The visual score for each class was calculated by multiplying the percentage of cells in the appropriate comet class by the value of the class. The degree of DNA damage was obtained by calculating the sum of scores in above mentioned five comet classes. Thus, the total visual score could range from 0 (all undamaged) to 400 (all maximally damaged) arbitrary units (AU), as reported by Collins et al. [37]. This method of measurement was proved to be valid and up-to-date [38]. Cell viability measured with trypan blue exclusion test was above 95% for all treatments. All experiments were repeated in triplicate.

#### 2.3.6. Detection of Apoptosis and Necrosis Using Acridine Orange/Ethidium Bromide (AO/EB) Staining

Sample preparation and cell culture

Sample preparation and cell culture is as mentioned in cytotoxicity assay. 

AO/EB staining

The applied method has previously described by McGahon et al. [39]. AO/EB solution was added to the cell suspension (1 part of 100 μg/mL of AO and 1 part of 100 μg/mL of EB in PBS) on a clean microscope slide and the nuclear morphology was evaluated by fluorescence microscopy (Leica DM 1000, Solms, Germany). Minimum 100 cells were counted and photos were taken at randomly selected areas. According to the method, live cells will appear uniformly green. Early apoptotic cells will stain green and contain bright green dots in the nuclei. Late apoptotic cells stain orange with condensed and often fragmented nuclei. Necrotic cells stain orange, but have a nuclear morphology with no condensed chromatin [40]. Tests were done in triplicate.

#### 2.3.7. Statistical Analysis

All outcomes including the measured cell viability using SRB and WST1, the Comet Assay and AO/EB staining evaluated statistically and the final results were expressed as Mean ± SD. The results are presented as tree replicates. Data in the experiments were analyzed using analyses of variance (One-Way ANOVA). Associations between anti genotoxic activity and oxidative stress parameters were analyzed by Pearson correlation coefficient. The *p* value < 0.05 was considered as statistically significant. All statistical analyzes were done by using SPSS package program for Windows (Version 11.5).

## 3. Results

### 3.1. Particle Size Distribution and Zeta Potential Assays

**Figure 2:**
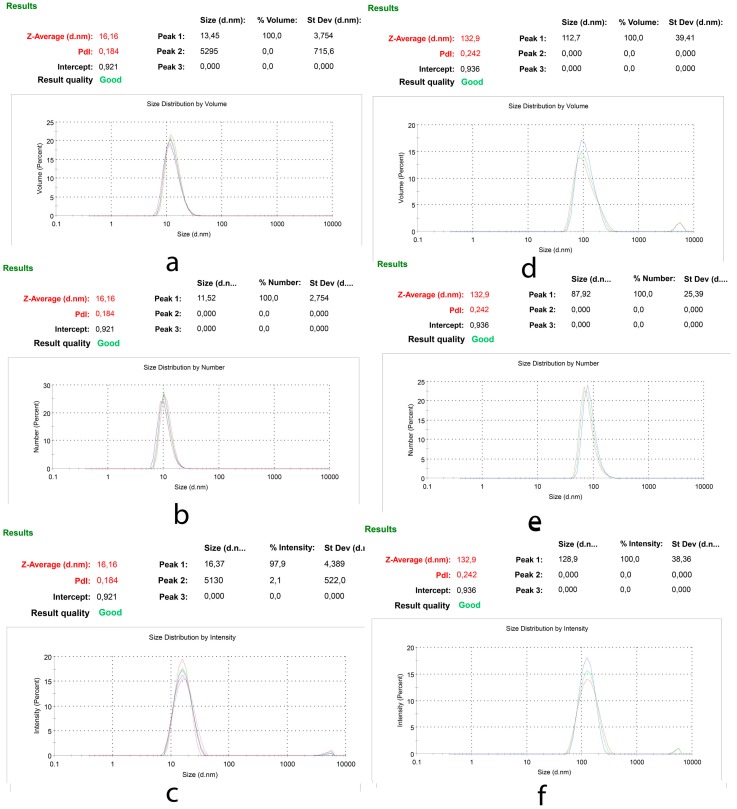
Particle size distribution of SSM and PM; (**a**) % Volume (**b**) % Number (**c**) % Intensity of SSM and (**d**) % Volume (**e**) % Number (**f**) % Intensity of PM.

Particle size measurements were used to evaluate the quality of produced nano-particles in PBS. Hydrodynamic size of SSM and PM was measured before each toxicity assay to ensure using uniform micelles with similar structures. Figure 2 shows the % volume, % number distribution and scattered intensity of SSM (with the Z Average = 16.16 nm) and PM (with the Z Average = 132.9 nm). Also, since in cell culture media we have an environment different than PBS and specially protein content of Complete Culture Media is an important factor in changing the size of nano-micelles, the size of these particles in Complete Culture Media was measured as well. The results obtained were much similar to that obtained in PBS. The particle size was in well accordance with our previous reports [13,14]. 

Table 2 presents the results obtained from Particle size and zeta potential assays.

Zeta potential of SSM and PM was measured as 0.01 and −26.3 mV in pH 7.7 PBS buffer respectively. These data are in complete agreement with previously reported surface charge of micelles made of PCL-PEG [41] and unpublished data recorded by our group in a master thesis [42] 

The size of SSM and PM in Complete Culture Media was measured to compare the particle sizes in cultured media. It is obvious that the toxicity of any particle is directly affected by its size. 

### 3.2. Cytotoxicity Assay

#### 3.2.1. SRB

**Figure 3:**
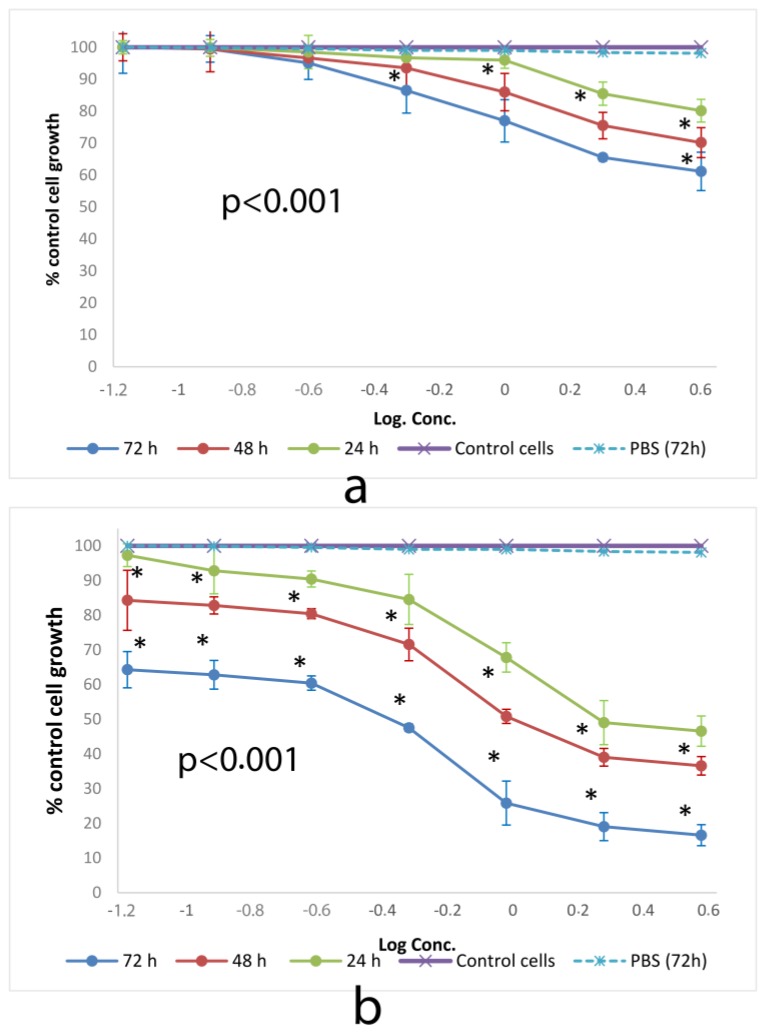
Cytotoxic effect of (**a**) SSM and (**b**) PM at 24, 48 and 72 h on L-929 cell lines determined by SRB assay. Data are representative of tree independent trials and are expressed as the mean ± SD. Significant differences between cell viabilities at 24, 48 and 72 h were indicated by * *p* < 0.05.

SRB method has been assigned as a more sensitive method than tetrazolium (MMT) assay with higher reproducibility and better linearity with the numbers of living cells. U.S. National Cancer Institute has described SRB method as a protein stain for in vitro chemosensitivity testing [43]. Figure 3 shows the cytotoxic effect of SSM and PM on L-929 cells at 24, 48 and 72 h of incubation determined by SRB assay.

All SSM concentrations showed cytotoxic activity with values above ED_50_ dose. However, PM showed ED_50_ values as 3.25, 1.38 and 0.29 mg/mL after 24, 48 and 72 h of incubation on L-929 cells respectively.

#### 3.2.2. WST-1

WST-1 (sodium salt of 4-[3-(4iodophenyl)-2-(4-nitrophenyl)-2H-5-tetrazolio]-1,3-benzene disulfonate) is an assay for measuring superoxide dismutase (SOD) activity. Changes in the activity of SOD involves in some pathologies because of its catalytic role in breakdown of superoxide radicals and defense against oxygen toxicity. Cells treated with different concentrations of micelles were treated with WST-1 to assess the activity of SOD as a detector of superoxide radical generated by xanthine oxidase and hypoxanthine [44].

**Figure 4:**
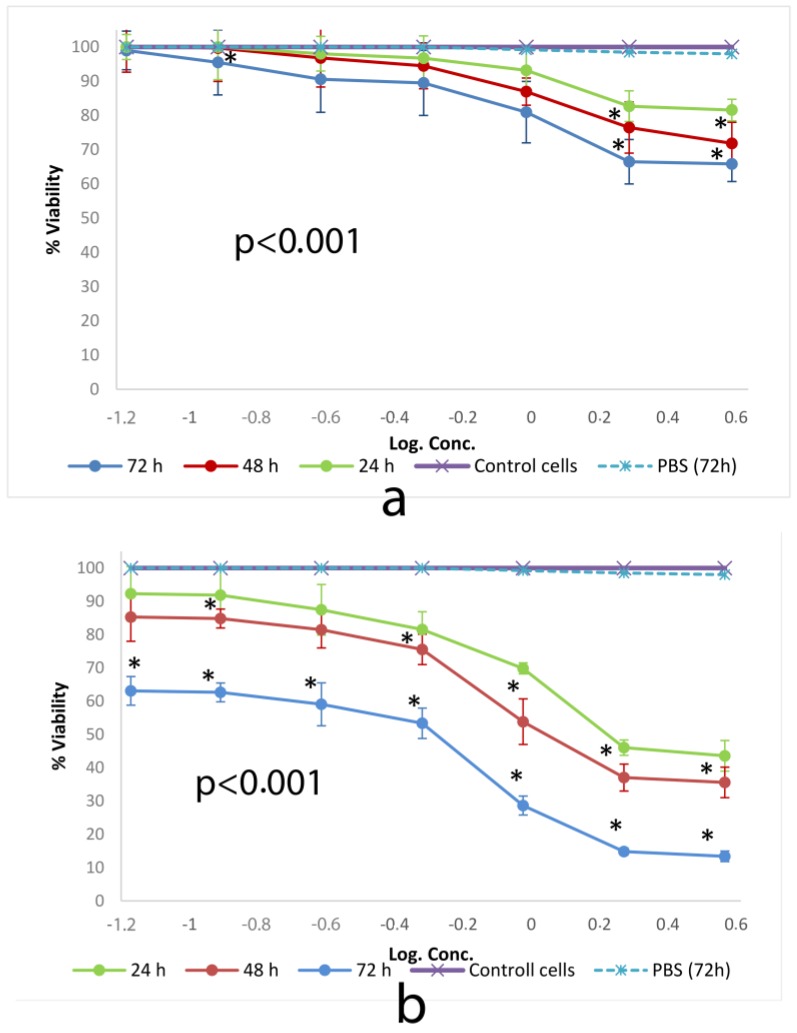
Cytotoxic effect of (**a**) SSM and (**b**) PM at 24, 48 and 72 h on L-929 cell lines determined by WST-1. Data are representative of tree independent trials and are expressed as the mean ± SD. Significant differences between cell viabilities at 24, 48 and 72 h were indicated by * *p* < 0.05.

Figure 4 shows cell viability ratios of cells incubated with different concentrations of SSM and PM as a consequence of SOD activity.

WST-1 test results were compatible with those of SRB. SSM did not show a cytotoxic activity to measure the ED_50_ value. The activity of PM showed ED_50_ values consisted of 2.8 mg/mL, 1.4 mg/mL and 0.29 mg/mL, after 24, 48 and 72 h of incubation on L-929 cells respectively. 

### 3.3. Genotoxic Activity Assays

At the International Workshop on Genotoxicity Test Procedures (IWGTP) held in Washington, DC, USA, 1999, an expert panel announced that the optimal version of the Comet assay for evaluating the possible genotoxic activity of different materials is the alkaline (pH = 13) version of the assay developed by Singh et al. at 1988 [35]. The pH 13 version is capable of detecting DNA single-strand breaks (SSB), alkali-labile sites (ALS), DNA-DNA/DNA protein cross-linking, and SSB associated with incomplete excision repair sites [45]. 

**Figure 5:**
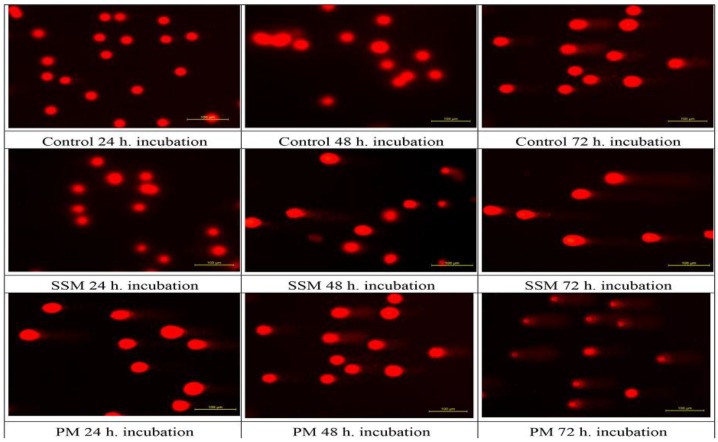
Genotoxic effect of 4 mg/mL of SSM and 1 mg/mL of PM on L-929 cells compared to non-treated ones after 24, 48 and 72 h incubation. Comet formation pattern showed that SSM induces a much lower DNA damage on these cells than PM after 48 and 72 h of incubation.

**Figure 6:**
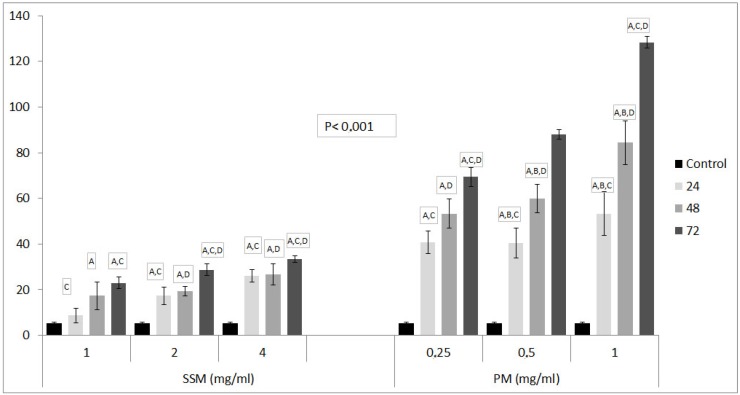
The comet assay results of SSM and PM on L-929 cell lines. Values are shown as Mean ± SD, which, are three separate experiments, performed in triplicate. Data are representative of tree independent trials and are expressed as the mean ± SD. Significant differences between DNA damage at 24, 48 and 72 h were indicated by *p* < 0.05 as: **A:** Significant differences between DNA damage of control and related Nano-micelles at indicated incubation periods, **B:** Significant differences between DNA damage of 24 h incubated cells and 48 h incubated cells, **C:** Significant differences between DNA damage of 24 h incubated cells and 72 h incubated cells, **D:** Significant differences between DNA damage of 48 h incubated cells and 72 h incubated cells with related Nano-micelles.

Figure 5 shows the fluorescence microscope images of DNAs of non-treated cells (Control), cells treated with 4 mg/mL of SSM and 1 mg/mL of PM at 24, 48 and 72 h. Figure 6, shows relative DNA damages caused by PM at three concentrations about ED_50_ values and at the highest concentrations of SSM used in cytotoxicity assays.

As it could be seen in Figure 5 and Figure 6 although both nano-micelles cause cell death at some concentrations, The DNA damage caused by SSM was significantly lower than that of PM. The DNA damage caused by incubation of 1mg/mL of PM with L-929 cells were striking.

### 3.4. Detection of Apoptosis and Necrosis Using Acridine Orange/Ethidium Bromide (AO/EB) Staining

**Figure 7:**
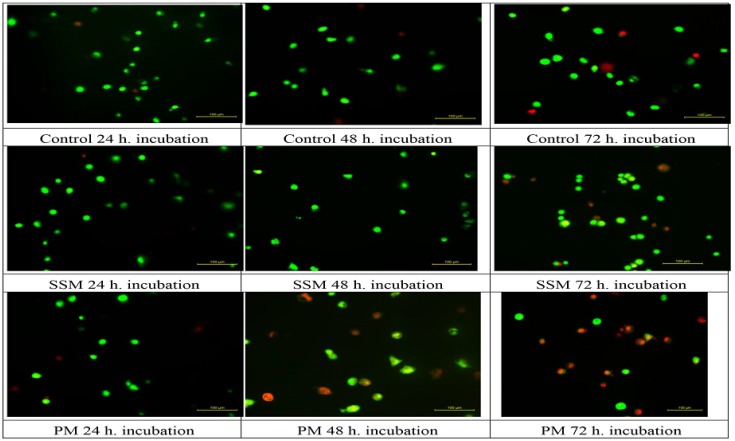
Different morphological patterns of cell death induced by 4 mg/mL of SSM and 1 mg/mL of PM on L-929 cells compared to non-treated ones after 24, 48 and 72 h incubation. Green live cells show normal morphology; green early apoptotic cells show nuclear margination and chromatin condensation. Late orange apoptotic cells showed fragmented chromatin and apoptotic bodies. Necrotic cells stain uniformly red.

**Figure 8:**
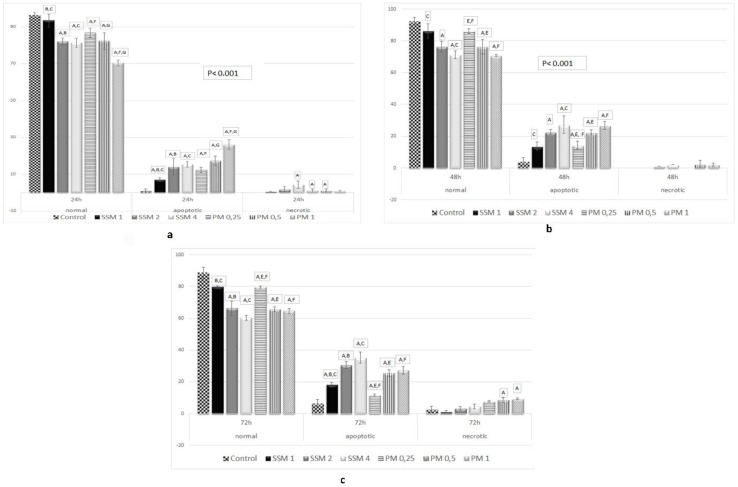
Apoptotic and necrotic activity of SSM and PM on L-929 cell lines. Cells were treated with different concentrations of SSM and PM for 24 (up, left, **a**), 48 (up, right, **b**) and 72 h (below middle, **c**), AO/EB double staining and measured by fluorimetery. There were positive correlations between cell viability ratios obtained from cytotoxicity assays and cell death (apoptosis + necrosis) ratios obtained from AO/EB assay. Significant differences between cell death ratios at 24, 48 and 72 h were indicated by *p* < 0.05 as: **A:** Significant differences between cell death at control and cells treated with related Nano-micelles **B:** Significant differences between 1 mg/mL and 2 mg/mL SSM, **C:** Significant differences between 1 mg/mL and 4 mg/mL SSM, **D:** Significant differences between 2 mg/mL and 4 mg/mL SSM, **E:** Significant differences between 0.25 mg/mL and 0.5 mg/mL PM, **F:** Significant differences between 0.25 mg/mL and 1 mg/mL PM, **G:** Significant differences between 0.25 mg/mL and 1 mg/mL PM.

AO permeates all cells and the nuclei become green whereas EB is only taken up by cells that their cytoplasmic membrane integrity is lost, and their nuclei are stained red. EB also dominates over AO. Thus, live cells will show a normal green nucleus. Early apoptotic cells should give bright green nucleus with condensed or fragmented chromatin. Late apoptotic cells display condensed and fragmented orange chromatin and necrotic cells have a uniform red appearance [46]. Figure 7 shows different morphological patterns of cell death induced by 4 mg/mL of SSM and 1 mg/mL of PM on L-929 cells compared to non-treated ones after 24, 48 and 72 h incubation. Figure 8 summarizes the cell death mechanism results obtained by AO/EB staining method.

As it could be seen in Figure 7 and Figure 8, the mechanism of cell death caused by incubating cells with SSM is only apoptosis (necrosis is ignorable), which, appears after 48 h and reaches to ≈% 35 after 72 h of incubation (≈% 60 normal cells). This is when, in case of incubating cells with PM the prevailing mechanism of cell death is apoptosis, however some necrotic cells were observed as well.

## 4. Discussion

Although the production technology of nano-engineered materials could still be counted as a young branch of science, there is fast evolution in quantity and quality of introduced materials to different eras of applications. The toxicity of these materials change by the route of contact with the biological system. There is no simple rule or formula to help the prediction and calculation of nano material toxicity considering all aspects of its physical interfaces or all biological properties of living organisms that are incorporated in the toxic behavior of nano material [8]. In general, materials chosen in the production of nano-particles for medical applications must be “bio-compatible” and “bio-degradable”. Bio-compatibility has been determined as the ability of a material to stay in contact with the human body without causing any unacceptable degree of harm to that body [47]. In the frame of NDDS studies biocompatiblity could be referred as: “(a) the ability to interact with the biological milieu in the absence of triggering acute adverse reactions (e.g., apoptosis, cell detachment, tissue necrosis); (b) naive immunoreactivity and absence of acute inflammatory responses; (c) absence of intoxication from metabolism of chemical components; (d) harmless or without long-term tissue accumulation leading to material deposits in the body” [48]. Biodegradability, however, is the ability of material to be degraded by natural pathways and leave the body within certain period. Non-biodegradable nano-materials can accumulate in tissues causing harmful side effects. In this case, the most important factor that affects the bio-degradability of a nano-material is the presence of different functional groups on its compartments and surfaces [48]. 

As mentioned in the Introduction part of this paper, EPR effect is the most important simple phenomenon for targeting cancer. However, fenestrated endothelium [49] appears in glands [50], digestive mucosa and the kidney as well [51]. This is how healthy cells become the “seconder target” of NDDS. To this point, it is important to evaluate cytotoxic and genotoxic effects of NDDS upon contact with healthy cells. Although biocompatibility requirements, such as absence of acute inflammatory responses, absence of intoxication from metabolism of chemical components and tissue accumulation, must be studied in vivo, adverse reactions such as apoptosis, cell detachment and tissue necrosis could be assessed prior to animal studies.

In this study, we studied cytotoxicity, mechanism of cytotoxicity and genotoxicity of lipid-based SSM and polymer-based PM. Particle size measurements were used to qualify the produced particles. SSM and PM showed hydrodynamic size of 16.16 and 132.9 nm (Z Average), which are the same as previously reported data [13,14]. Because of their size, clearly therapeutic agents delivered in SSM will be subject to more efficient EPR effect and better targeted to tumors, when compared to drugs delivered in PM. Obviously the PEG layer surrounding both nano-micelles prevents adherence of the proteins of complete culture media on micelles, which is clearly seen by the results obtained from particle sizing.

Liposomes are the most studied lipid-based drug delivery systems, because they are the only FDA approved NDDS so far. It has been shown that the toxicity of liposomes is correlated with their surface charge. The toxicity arising from surface charge is so important that liposomes with positive surface charge are useless clinically. Cationic liposomes can interact with serum proteins, lipoproteins, and the extracellular matrix, leading to aggregation or release of loaded agents before reaching the target cells, leading to systemic toxicity [52,53,54]. Also, previous studies have shown that quaternary amines are more toxic and inhibit PKC (Protein Kinase C) activity (an indicator of transfection efficiency) compared to tertiary amines [55]. Although 1,2-distearoyl-*sn*-glycero-3-phosphatidylethanolamine-*N*-[methoxy (polyethylene glycol)-2000] (DSPE-PEG2000) is a negatively charged particle due to the PEG shell, SSM is a neutral particle [56]. As it could be understood from above mentioned chemical name, it also carries tertiary amine [15]. These two facts are evidence that show SSM is a biocompatible particle. Furthermore, DSPE-PEG 2000 has previously been used in formation of Liposomal Doxorubicin (Doxil), which is now widely used in clinic. Previously, camptothecin loaded SSM has been used in targeted therapy of rheumatoid arthritis [23]. In this study, a single subcutaneous injection of 0.1 mg/kg of camptothecin loaded SSM has significantly mitigated collagen-induced arthritis in mice for 32 days. During this long period, no systemic toxicity has been evidenced on tested animals. According to our best knowledge, this is the first report on the cytotoxicity of SSM on healthy fibroblast cells in vitro. Our results support all previous evidence of good toxicity profile of SSM [15,57,58]. None of the concentrations studied showed cytotoxicity effect on L-929 cells, either on SRB or on WST-1 tests. This is a valuable result because SRB measures the protein amount of living cell, while WST-1 indicates the functionality of the cell, because only the living cell is able to reduce tetrazolium salt to a soluble formazan salt by a reductase of the mitochondrial respiratory chain [43,44]. 

It is noteworthy that the concentrations chosen in this study are much higher than the concentrations needed to carry the therapeutic dose of drug molecules in vivo. In our previous study the amount of SSM to carry the effective dose of Vinorelbine (1.2 µg Vinorelbine) was established as 0.0067 mg/mL. In this study, even the lowest concentration of SSM applied to cells (0.0625 mg/mL) is almost ten times higher than this amount that showed no toxicity event at the end of 72 h of incubation. The highest concentration of this lipid-based NDDS applied to cells was 4 mg/mL, which showed %20, %30 and %39 toxicity at the end of 24, 48 and 72 h of incubation. Considering the results obtained from cytotoxicity assays of SSM together with the DNA damage results caused by this material shows that DNA damage does not play a significant role in the low cytotoxicity of SSM. AO/EB staining assay showed that the mechanism of cell death in these concentrations was apoptosis. 

The occurrence of apoptosis without associating with DNA damage is a known phenomenon in case of NDDS studies. Nano-particles are frequently detected in lysosomes upon internalization, and a variety of nano-materials have been associated with lysosomal dysfunction [59,60]. 

The polymeric materials used in this study consisted of PCL and PEG. Both materials are well known biocompatible polymers [61]. Previously, cytotoxicity of these materials have been evaluated using PEG-PCL-PEG self-assembly systems [62]. In this study, PCL forms the hydrophobic inner core and PEG chains fold out, to create the hydrophilic outer shell. This structure is very similar to the structure used in the currently reported study, except that we used di-block copolymers, not the three-block one. The cytotoxicity results they have obtained are in a good correlation with our results. Both studies are reporting significant toxicity (48% cell death reported previously, 53% cell death reporting now) effect on L-929 cells [62] caused by 4 mg/mL of polymeric material after 48 h of incubation. The percent viability measured by our group at the end of 72 h of incubation was 16% (84% cell death). Also there is a significant DNA damage caused by PCL-PEG at the end of 72 h. The mechanism of cell death caused by PM has been established to be apoptosis using AO/EB staining assay. Some necrotic cells were evidenced in case of inoculation of cells with PM for 72 h. In our previous data the amount of PM used to carry the therapeutic amount of Vinorelbine (1.96 µg Vinorelbine) was calculated as 0.028 mg/mL [13]. Again in this study the lowest concentration of polymeric material treated with L-929 cells (0.0625 mg/mL) is almost three times higher than this concentration.

The DNA mechanism of damage caused by “some” nano-particles has been well established. This damage starts with entry of NDDS to the cell. Entry of NPs into a cell is largely governed by biological mechanisms of endocytosis [63]. The best known of these are clathrin-mediated endocytosis and caveolin-mediated endocytosis [64,65]. All these endocytic routes of uptake involve delivery of NDDS into a subcellular compartment, that is, the endosome. Most of these endocytic routes also end up in a degradative compartment of the cell, that is, the lysosome, where materials are exposed to high concentrations of a wide variety of hydrolytic enzymes. Upon internalization, the NPs may presumably be degraded into ions in the lysosomes. These “free ions” can potentially pass through the nuclear or mitochondrial membrane and in the latter case react with hydrogen peroxide (H_2_O_2_) and oxygen produced by the mitochondria to produce highly reactive hydroxyl radicals. Hydroxyl radicals (^−^OH) generated could indirectly damage DNA, which eventually ends up with apoptosis [66]. 

The mechanism of necrosis is well defined as well. Polymeric materials, in contrast to lipid-based NDDS, are not able to integrate with the phospholipid structure of cell membrane. We hypothesize that incubation of polymeric NDDS with cells cause disruption of their cell membrane. It is well-known that the key mechanism of necrosis is cell membrane damage. The initial alterations of cellular metabolism and electrolyte homeostasis induced by an injurious agent may activate at least four major pathways leading to loss of membrane integrity: membrane phospholipid degradation, production of amphipathic lipids, damage to the cytoskeleton, and generation of toxic oxygen species and free radicals [67]. These possibilities could point the mechanism of action of PM in resulting necrosis in L-929 cells.

## 5. Conclusions

The cell toxicity of lipid-based micelles (SSM) against L-929 cells are much lower than that of Y-shaped PCL_2_-PEG polymeric material. The mechanism of cell death has been assessed as apoptosis in case of applying both SSM and PM. Some necrotic cells were observed in the case of PM. Similarly, the DNA damage caused by SSM is ignorable compared to that of PM. We can conclude that the integration of nano-materials with the phospholipid structure of the cell membrane is one major reason for the better toxic profile of SSM. In case of PM, however, either the synthetic nature of the materials or the chemicals used in click process of synthesis are responsible of observed toxicity. It is known that the click process of phospholipids to PEG consists of a simple esterification step, which, possibly provides less toxicity for obtained amphiphilic material. 

The particle sizes and zeta potentials of studied micelles are different and this could affect their biological properties, such as the ability of being uptaken by cells. We previously reported that toxicity of Vinorelbine, a very well-known chemotherapeutic agent, shows enhancement by loading to the studied micelles [13,14]. In those studies, we also showed that the empty micelles have no cytotoxic effect on MCF-7 breast cancer cell lines. Compering those studies with our currently presented one, in which empty micelles are used, clearly shows that the toxic effects obtained in this manuscript are the consequences of endocytosis of the micelles into the cells. It is noteworthy that the concentrations used in this study to evaluate the healthy cell toxicity are far higher than what is being used to carry the therapeutic amounts of drug molecules. As we previously have reported SSM and PM are used as 0.0067 and 0.028 mg/mL respectively to carry the therapeutic amounts of chemotherapeutic drugs [13,14]. Thus, the reported toxic effects must be considered only if these nano-micelles accumulate in a certain organ or tissue. Previously, it has been shown that the accumulation of nano-particles in different body organs is size dependent, but they accumulate at almost all organs including liver, spleen, kidneys, testes, thymus, heart, lungs and brain. Thus, the toxicity values obtained in this research are precious in case of long-period contact of studied materials with cells such as liver and spleen [68].

Since SSM is a well-studied nano-drug delivery system, and no systematic toxicity was observed during in vivo investigations, further evaluations of in vivo toxicity and bio-distribution are needed to understand the behavior of Y-shaped PM in vivo. 

## Figures and Tables

**Table 1:** ^1^H-NMR chemical shifts and multiplicities of the functional groups of PEG-PCL_2_ in CDCl_3_, δ.

Functional Groups	Chemical Shifts	Multiplicities
**Ar*H* of anthracene**	8.30	d *
**Ar*H* of anthracene**	8.03	d *
**Ar*H* of anthracene**	7.60–7.47	m *
***CH*_2_–anthracene**	6.2	s *
**C*H*_2_OC=O of PCL**	4.05–4.02	br *
**C*H*_2_OH of PCL end-group**	3.60	bs *
**C=OC*H*_2_ of PCL**	2.20	br *
**C*H*_2_C*H*_2_C*H*_2_ of PCL**	1.80–1.20	m *

d *: doublet, m *: multiplet, s *: singlet, br *: broadened, bs *: broadened singlet.

**Table 2:** Particle size distribution of SSM and PM in PBS and in Complete Culture Media (nm).

Media	PBS								Complete Culture Media
	% Volume	SD	% Number	SD	% Intensity	SD	PDI	Zeta Potential (mV)	% Intensity
**SSM**	13.45	3.754	11.62	2.754	16.37	4.389	0.194	0.01	16.25
**PM**	112.7	39.41	87.92	25.39	128.9	38.36	0.242	-26.3	132.56
